# Development and evaluation of an interactive web-based decision-making programme on relapse management for people with multiple sclerosis (POWER@MS2)—study protocol for a randomised controlled trial

**DOI:** 10.1186/s13063-021-05059-1

**Published:** 2021-02-14

**Authors:** Anne Christin Rahn, Lisa Wenzel, Andrea Icks, Alexander Stahmann, Jutta Scheiderbauer, Kristina Grentzenberg, Markus Vomhof, Joseph Montalbo, Tim Friede, Christoph Heesen, Sascha Köpke

**Affiliations:** 1 Institute of Neuroimmunology and Multiple Sclerosis, University Medical Center Hamburg-Eppendorf, Hamburg, Germany; 2 Department of Health Services Research, Carl von Ossietzky University Oldenburg, Oldenburg, Germany; 3 Institute of Nursing Science, University Hospital Cologne and Faculty of Medicine, University of Cologne, Cologne, Germany; 4 Institute for Health Services Research and Health Economics, Centre for Health and Society, Heinrich Heine University Düsseldorf, Düsseldorf, Germany; 5 MS Forschungs- und Projektentwicklungs-gGmbH, Hannover, Germany; 6 Stiftung für Selbstbestimmung und Selbstvertretung von MS-Betroffenen, Trier, Germany; 7 Department of Medical Statistics, University Medical, Göttingen, Germany; 8 Department of Neurology, University Medical Center Hamburg-Eppendorf, Hamburg, Germany

**Keywords:** Multiple sclerosis, Decision-making, Relapse, Decision aid, Patient empowerment, Randomized controlled trial

## Abstract

**Introduction:**

Multiple sclerosis is a chronic inflammatory, degenerative disease of the central nervous system manifesting at first with relapses in about 85% of cases. In Germany, intravenous therapy with high-dose corticosteroids is the treatment standard of acute relapses. The treatment leads to a faster reduction of symptoms in about 25 of 100 treated patients but has no proven long-term benefits over placebo treatment. Intravenous treatment is not superior to oral treatment. Therefore, informed decisions on relapse management are required. An earlier randomised controlled trial showed that evidence-based patient information and education on relapse management leads to more informed decisions and more relapses not treated or treated with oral corticosteroids. This study aims to evaluate whether a web-based relapse management programme will positively change relapse management and strengthen autonomy in people with multiple sclerosis.

**Methods:**

The pragmatic double-blind randomised controlled trial is accompanied by a mixed-methods process evaluation and a health economic evaluation and follows the UK Medical Research Council guidance on developing and evaluating complex interventions. A total of 188 people with possible or relapsing-remitting multiple sclerosis with ≥ 1 relapse within the last year and/or ≥ 2 relapses within the last 2 years will be recruited and randomised using blocks. The intervention group receives a web- and dialogue-based decision aid on relapse management, a nurse-led webinar and access to a monitored chat forum. The control group receives standard information, which will be made available via the same online platform as the intervention. The primary endpoint is the proportion of relapses not treated or treated with oral corticosteroids. Key secondary endpoints are the annualised relapse rate, decision-making, empowerment, quality of life and cost-effectiveness. Facilitators and barriers will be assessed by mixed-methods process evaluation measures. The study ends when 81 relapses have been documented or after 24 months of observation per individual patient. Analyses will follow the intention-to-treat principle.

**Discussion:**

We hypothesise that the intervention will enhance patient empowerment and have a positive impact on patients’ relapse management.

**Trial registration:**

ClinicalTrials.govNCT04233970. Registered on 18 January 2020

## Administrative information

The order of the items has been modified to group similar items (see http://www.equator-network.org/reporting-guidelines/spirit-2013-statement-defining-standard-protocol-items-for-clinical-trials/).

**Table:** 

Title [1]	Development and evaluation of an interactive web-based decision-making programme on relapse management for people with multiple sclerosis – a randomized controlled trial protocol (POWER@MS2)
Trial registration {2a and 2b}.	ClinicalTrials.gov; NCT04233970, registered on the 18th of January 2020
Protocol version {3}	2.3, 14th of January 2021
Funding {4}	This investigator-initiated study is publicly funded by the German Innovation Fund (Innovationsfonds, 01VSF 17015) of the Federal Joint Committee (Gemeinsamer Bundesausschuss, G-BA).
Author details {5a}	Anne C. Rahn (AR) Department of Health Services Research, Carl von Ossietzky University Oldenburg, Oldenburg, Germany K. Grentzenberg (KG) and Christoph Heesen (CH): Institute of Neuroimmunology and Multiple Sclerosis, Medical Centre Hamburg-Eppendorf (UKE), Hamburg, Germany Sascha Köpke (SK): Institute of Nursing Science, University of Cologne, Cologne, Germany Lisa Wenzel (LW): Institute of Nursing Science, University of Cologne, Cologne, Germany; Institute of Neuroimmunology and Multiple Sclerosis, Medical Centre Hamburg-Eppendorf (UKE), Hamburg, Germany Tim Friede (TF): Department of Medical Statistics, University Medical Center Göttingen, Göttingen, Germany Andrea Icks (AI), Markus Vomhof (MV). Joseph Montalbo (JM): Institute for Health Services Research and Health Economics, Centre for Health and Society, Medical Faculty, Heinrich Heine University Düsseldorf, Germany Jutta Scheiderbauer (JS): Stiftung für Selbstbestimmung und Selbstvertretung von MS-Betroffenen, Trier, Germany Alexander Stahmann (AS): MS Forschungs- und Projektentwicklungs-gGmbH, Hannover, Germany
Name and contact information for the trial sponsor {5b}	Medical Centre Hamburg-Eppendorf (UKE), Hamburg, Germany Prof. Dr Blanche Schwappach-Pignataro +49 40 7410 52003, dekanin@uke.de
Role of sponsor {5c}	The funding body (German Innovation Fund of the Federal Joint Committee) is not involved in and has no influence on any study related aspect as for example study design, management, analysis, interpretation and dissemination of study results.

## Introduction

### Background and rationale {6a}

Multiple sclerosis (MS) is an inflammatory and degenerative disease of the central nervous system, which affects about 200,000 mostly young people in Germany. MS manifests initially with relapses in about 85% of cases [2–4]. The estimated cumulative incidence for 2015 in Germany was 18 new cases per 100,000 persons [3]. Due to the chronic course of the disease over decades and the restrictions in activity and participation on the one hand, as well as the continuous approvals of new high-price immunotherapy options, on the other hand, MS is of high health economic relevance [5]. Despite a lack of evidence, intravenous (IV) inpatient therapy of MS relapses with high-dose corticosteroids is the dominating management approach in Germany [6]. The German guideline on MS management is currently under revision and it is expected that the new recommendations will reflect the evidence showing that IV corticosteroids are not superior to oral administration [7, 8]. International guidelines recommend oral corticosteroid administration as the first treatment choice [9]. Currently, IV corticosteroid therapy for relapses is one of the main reasons for hospital admissions of people with MS (PwMS) in Germany [10], while only 36% of PwMS underwent outpatient relapse treatment between 2006 and 2011 [11]. The direct and indirect costs of an MS relapse in Germany amount to approx. €3000 [12]. In some metropolitan areas, structured patient information programmes are available, e.g. at the University Hospital in Hamburg aiming at informed decision-making in PwMS [13]. Noteworthy, Hamburg has the lowest hospitalisation rate of PwMS in Germany [14]. Provision of information and education programmes in rural areas is challenging. Therefore, the “White Paper Multiple Sclerosis” calls for intelligent care concepts to counteract the undersupply of PwMS, especially in rural areas [15].

eHealth technologies or telehealth concepts, which include online training and education programmes, hold the potential for a better, more easily accessible and cheaper infrastructure in health care [16, 17]. An interactive web-based relapse management programme could provide access for PwMS in rural areas. Results of a multicentre survey amongst PwMS in Germany showed that 94% of the 586 respondents have internet access, regardless of whether they live in rural or urban areas [18]. PwMS belong to the group of patients with high internet affinity [19–21], and they accept online programmes well [22–24]. A recent Cochrane Review on telerehabilitation in MS included nine RCTs [25]. In five studies, the intervention took place via the internet. The authors concluded that there is limited evidence of the efficacy of telerehabilitation in improving functional activity, quality of life and fatigue. The review also showed that there is no RCT with an economic evaluation and little evidence on programme satisfaction from process evaluations [25]. So far, there are no studies in which online education programmes on steroid therapies for PwMS have been evaluated [26].

According to the German patients’ right act, patients need to be informed about the treatment options including the possible benefits and risks [27]. Evidence-based patient information (EBPI) [28] and patient decision aids [29] are important facilitators of informed decision-making. EBPI is based on a transparent methodological procedure by considering the current scientific evidence. It provides comprehensive, understandable, transparent, unbiased and objective information on health decisions [28, 30]. Patient decision aids are based on EBPI, taking into account personal values and preferences [31], and have been shown to improve knowledge regarding options and reduce decisional conflict [29].

A randomised controlled trial (RCT) including 150 PwMS showed that an evidence-based relapse management group training programme with critical consideration of steroid therapy leads to a more differentiated use of steroids without negative consequences for quality of life or disease progression [32]. In the intervention group, more relapses were treated with oral steroids or were not treated (108/139 (78%) intervention group vs. 101/179 (56%) control group, difference 22%, 95% CI 11–31%)) and autonomous treatment decisions were more frequent (difference 27%, 95% CI 16 to 37%). Also, fewer relapses occurred over 2 years (mean (SD) number of relapses 1,9 (1,6) intervention group vs. 2,7 (2,1) control group, 95% CI - 1.4 to - 0.1) [32]. A subsequent implementation study has shown that the transfer to other settings is possible but has important challenges and limitations [33].

In POWER@MS2, we will build on this evidence by transferring the content to a web platform and making the intervention easily accessible and implementable. Therefore, the programme aims to increase the participation of PwMS in decision-making processes, reduce the burden of physicians and improve relapse management.

## Objectives {7}

We hypothesise that the web- and evidence-based relapse management programme POWER@MS2 results in more autonomous relapse treatment decision-making by PwMS. Therefore, we investigate whether a web-based training on self-management of relapses based on an evaluated but hardly implemented group training programme in PwMS [32] can be successfully and effectively implemented.

### Primary objective

We aim to demonstrate that POWER@MS2 leads to fewer relapses treated with corticosteroids and, in case of steroid treatment, less IV and more orally administered corticosteroids (primary endpoint: the proportion of relapses not treated or treated with oral corticosteroids).

### Secondary objectives

The secondary aims are to determine if POWER@MS2:

- Results in more autonomous relapse treatment decisions
- Leads to fewer relapses
- Leads to increased risk-knowledge
- Leads to an increased sense of control
- Leads to more empowerment
- Is cost-effective

Furthermore, the fit between the technology (web-based programme), users and context factors will be explored by a process evaluation addressing fidelity and dose as facilitators and barriers. A protocol describing the process evaluation methods will be published separately.

## Trial design {8}

**Fig. 1:**
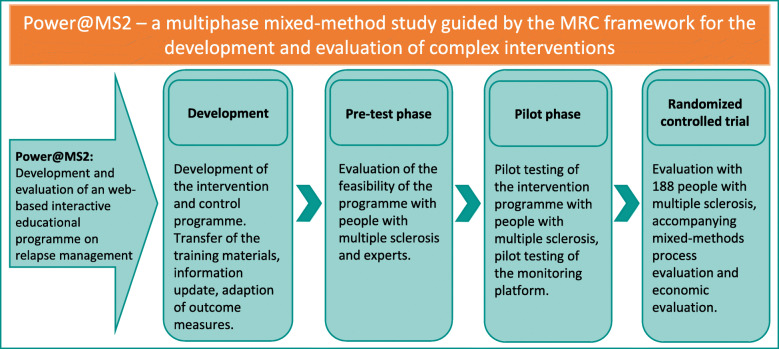
Study design POWER@MS2

POWER@MS2 will be carried out as a “multiphase mixed-methods study” following the Medical Research Council (MRC) framework for the development and evaluation of complex interventions [34, 35] (see Fig. 1). This study design is suitable for the development and evaluation of complex interventions and is also recommended in the context of eHealth interventions [16]. The study will follow the concepts of EBPI [28]/patient decision aids [36] and empowerment [37]. The Theory of Planned Behaviour will be applied as a health behaviour model [38].

The project will be conducted by a multidisciplinary team with close collaboration between the creators of the web-based programme (physicians, health scientists, nurses, health economists, statisticians, graphic designers, psychologists and programme developers) and stakeholders to ensure usability and need-based orientation. The stakeholders are PwMS, patient representatives (German Multiple Sclerosis Society (DMSG)) as well as clinicians and MS experts.

**Fig. 2:**
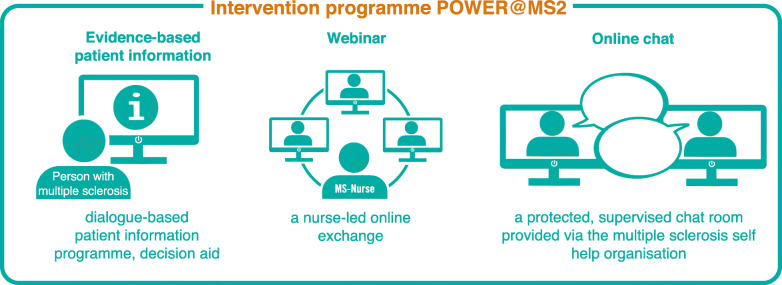
Complex intervention components POWER@MS2

This study protocol focuses on the evaluation by a RCT. The process evaluation will be described in an additional publication. The content of all project phases is described shortly in the following (Fig. 1):

- *Development*: The original training programme consisted of a 40-page EBPI on relapse management and 4 h of interactive group training, facilitated by an MS nurse and an expert patient. We updated the content of the EBPI by systematic literature searches and developed the web-based programme guided by the original training programme materials. The intervention is designed as an individualised, dialogue-based system that provides PwMS with coordinated individually tailored information based on the artificial intelligence (AI)-based software platform broca®. Broca® built the foundation for several effective therapy support systems [23, 24]. A nurse-led webinar offers a structured exchange and a protected chat room allows further interaction (see Fig. 2).
- *Feasibility and piloting*: To test the feasibility [39], we presented the web-based programme to patient representatives, PwMS and MS experts in the pre-test phase. We explored several aspects of feasibility such as practicability and acceptance. Further, we tested the webinar with selected PwMS.
  In the next step, we piloted the revised programme with 7 PwMS to test comprehensibility, usability, accessibility and acceptance of the programme including the webinar and the chat room. Three experts reviewed the EBPI. Furthermore, we tested user-friendliness, accessibility, time to fill in the questionnaires and the monitoring platform in the MS register. Based on the pilot testing, we finalised the programme. The participants of the pre-test and pilot phase will be excluded from the main study.
- *Evaluation:* The intervention will be evaluated in a pragmatic parallel-group, superiority, double-blind (PwMS and outcome assessors) RCT. Participants will be randomised to the web-based programme (intervention group) or a standard information on relapse management based on the contents of the German Self-Help Organisation DMSG (control group). In addition, a mixed-methods process evaluation and a health economic evaluation will be carried out.

## Methods: participants, interventions and outcomes

### Setting, recruitment and procedure

#### Study setting {9}

PwMS will be recruited via all participating centres (private neurological practices and MS outpatient departments of academic and community hospitals) in Germany. In the federal state of Schleswig-Holstein, all neurologists will be contacted.

#### Eligibility criteria {10}

##### Inclusion criteria

*Study centre eligibility criteria*

Neurological practices, as well as academic or community centres located in Germany, will be eligible to participate as study centres.

*Patient inclusion and exclusion criteria*

PwMS aged 18 to 65 years with suspected or diagnosed relapsing-remitting MS [40] with ≥ 1 relapse in the last year and/or ≥ 2 relapses in the last 2 years will be included. As the intervention will be offered online, only PwMS from one of the participating centres and with access to the internet can be included in the study. Only PwMS who are fluent in German and provided signed informed consent will be included.

PwMS with primary or secondary progressive MS will be excluded. PwMS with an acute relapse as well as those allergic to steroids will be excluded. Severe cognitive deficit, severe visual impairment or severe psychiatric disorder (based on clinical impression), which hinder information uptake and completion of the questionnaires, will be the reasons for exclusion. Further, PwMS who participate in the parallel study POWER@MS1 (https://clinicaltrials.gov/ct2/show/NCT03968172) or participated in the former training programme on relapses [32] will be excluded.

#### Who will take informed consent? {26a}

Interested and eligible PwMS will be provided with a study information sheet by the participating MS centres. Informed consent will be obtained by a physician in the MS centre.

#### Additional consent provisions for collection and use of participant data and biological specimens {26b}

Participants can also provide consent to participate in the national MS registry led by the DMSG and thus enable follow-up even beyond the end of the study. Informed consent will be obtained by a physician in the MS centre.

### Interventions

#### Explanation for the choice of comparators {6b}

We choose an optimised standard intervention to reflect current practice, while also trying to keep participants blinded. Therefore, we provide standard information of the German Self-Help Organisation on relapse management via the same platform as the intervention.

#### Intervention description {11a}

##### Intervention group (IG)

POWER@MS2 is designed as a highly individualised, dialogue-based system that provides PwMS with coordinated information based on their existing knowledge, interests, etc., based on the AI-based software platform broca® (see section “design”). Organised by the multidisciplinary research team, PwMS and MS experts, as well as patient representatives, played a central role in the design of content, style and format.

As outlined above, the intervention is based on an evaluated group training programme on relapse management with an EBPI [32]. Therefore, the original content of the EBPI and the group training were transferred to the broca® platform and updated as well as adapted.

While the group training programme was based on the “Protection Motivation Theory” [41], the current intervention is based on the comparable “Theory of Planned Behaviour” [38], which had been applied in studies on immunotherapy treatment decision-making of the working group [42, 43]. The concept of patient empowerment [37] lays the foundation of the intervention as well as of the theory application in this study. Therefore, the programme is guided by the self-determination theory as an underlying principle of empowerment [44]. In line with the empowerment concept, the content imparted by the web-based programme is based on the principles of EBPI [28, 30]/patient decision aids [39] and the knowledge transfer reflects educational concepts [45]. Based on current research and theory on eHealth, the programme follows the principles of responsiveness [46] and individual content-tailoring [47, 48] to reach a change of behaviour in PwMS based on educational knowledge provision. Therefore, the programme uses a variety of techniques such as information provision, weighing of pros and cons, action planning, preparing for and dealing with relapses, using prompts and reminders, modelling, constructing plans and formulating implementation intentions with the application of cognitive behavioural therapy techniques, e.g. behavioural activation. The intervention specifically attempts to avoid fear appeals and simple information provision (e.g. “lecturing”). In addition, e-mail reminders will be used to enhance involvement.

The web-based programme consists of three components (Fig. 2):

- EBPI with decision aid (five modules and a decision aid in case of an acute relapse, a menu section with summaries, videos, audios and a relapse report) provided by the broca® programme. The key element of the EBPI is the information on corticosteroids for the treatment of acute relapses. The programme will support the participants in treatment decision-making on relapses but does not take over decision-making and does not advice participants about what to do. The modules will be gradually activated over 4 weeks. A reminder system with neutral e-mail reminders will be used to promote the use of the programme. Non-users will receive a telephone call from the study nurse.
- A webinar led via Cisco WebEx (https://www.webex.com/de/index.html) by a trained MS nurse with a questioning/chat session (approx. 60–75 min). Participants who do not take part in the webinar will be contacted by the study nurse to encourage participation.
- A supervised chat room provided via the DMSG (www.msconnect.de).

The programme will be activated when approximately 10–14 participants have been recruited, so that a webinar can take place for this group. However, the waiting time until the start of the programme should not exceed 4–6 weeks. The programme can be used by the participants also after the webinar, e.g. in case of a relapse. Participants will have access to the programme for up to 2 years.

Upon completion, participants will receive a training certificate based on a successfully completed multiple-choice knowledge questionnaire integrated in the programme as well as an information sheet on steroids focusing on possible side effects. In principle, treatment with oral methylprednisolone (500–1000 mg for 3–5 days based on individual physician prescription decision) should be made available for participants after the training. Participants should be able to obtain the prescription from the neurologist after the presentation of the certificate, provided that the physician has no objections about the patient taking the medication on their own. Both the patient/participant and the physician can refuse oral self-medication, which does not change the course of the study.

##### Control group (CG)

Participants in the CG will have access to web-based information material offered via the same platform (broca®) in addition to usual care. The CG intervention will be based on materials of the DMSG on relapse management. The entire content of the programme can be accessed at once and a reminder system with neutral e-mail reminders will be used to promote the use of the programme.

#### Criteria for discontinuing or modifying allocated interventions {11b}

Participants can leave the study at any time and may withdraw consent. There will be no special criteria for discontinuing or modifying allocated interventions.

#### Strategies to improve adherence to interventions {11c}

To ensure involvement and adherence of participating MS centres, CH, LW and AR will provide interested MS centres with comprehensive information about the study including a promotional information video. Study site selection phone calls aim to establish early trial conduct fidelity and participating centres are invited to take part in the yearly study group meetings during the Annual German Neurological Society Conference.

To ensure adherence of PwMS to the intervention and control programme, all participants will be contacted through regular e-mail reminders by the programme. Participants in both groups will be contacted by phone every 3 months. Usage of the programmes will be monitored. In case of non-use of the intervention programme or other emerging difficulties, study participants will be additionally contacted by a member of the coordinating centre via e-mail or telephone.

#### Relevant concomitant care permitted or prohibited during the trial {11d}

In the case of, e.g., a relapse during the study, the participant is free to consult her/his neurologist and receive appropriate treatment.

#### Provisions for post-trial care {30}

There is no anticipated harm. No compensation for trial participation will be provided (see also {22}).

### Outcomes {12}

#### Primary outcome

The primary endpoint is the proportion of untreated or orally treated relapses during the follow-up of at least 12 and at most 24 months. The evaluation of relapses is based on a self-report of PwMS assessed by standardised questions during the 3-monthly telephone interviews, which has been successfully applied previously [32]. Two blinded MS experts (neurologists) will independently rate all relapses at the end of the study as definite relapses, possible relapses or no relapses based on the information assessed in the 3-monthly telephone interviews.

#### Secondary outcomes

Key secondary endpoints include relapse rates, decision autonomy, empowerment, quality of life, process evaluation measures and an extensive economic evaluation. It is expected that the programme will lead to the empowerment of participants and a more autonomous steroid treatment decision-making, to an increased risk knowledge, fewer relapses and a higher sense of control. No negative consequences on quality of life, disability progression, or anxiety and depression are expected.

The annualised relapse rate will be calculated based on the standardised assessment of relapses during the 3-monthly phone interviews. Further, relapses will be assessed in more detail using a questionnaire with 24 items based on the Hamburg Relapse Assessment Scale (HARAS) [49].

Risk knowledge on relapses will be assessed by the questionnaire used before [32], which has been slightly adapted to reflect current evidence. The questionnaire consists of 10 questions (9 multiple choice questions and one free-response question) with higher scores indicating better knowledge (score range 0–11).

Behaviour strategies in case of relapses will be measured by the validated Planned Behaviour in MS Scale (PBMS) [50], which had been developed for immunotherapy decision-making in MS and has now been adapted to steroid decision-making (PBMS relapse). The new questionnaire consists of 18 items covering the three domains “attitude”, “subjective social norm” and “control beliefs”. Within each domain, every item is classified to either “expectations” or “values” resulting in 6 subdomains.

Preferred and realised role preferences in steroid treatment decision-making will be assessed based on the Control Preference Scale (CPS) [49]. The CPS consists of five cards illustrating roles ranging from A (the individual making the treatment decisions), over C (the individual making the decisions jointly with the physician) to E (the physician making the decisions). The web-based version of the CPS card set has been validated, showing satisfactory results concerning reliability in MS [51]. Satisfaction with the decision will be measured in a telephone interview within 3 months in case a decision for or against steroid treatment has been made.

Empowerment will be assessed using 10 adapted items of the Patient Activation Measure (PAM13) [52], which demonstrated to be reliable and valid in an MS sample [53] as well as an adapted empowerment scale with 5 items from Bann et al. [54].

Impairment will be assessed by the neurologist at baseline and after 12 months using the Expanded Disability Status Scale (EDSS) [55] as a moderator variable. Furthermore, the self-reported United Kingdom Neurological Disability Scale (UNDS) will be used to assess impairment at baseline and from months 12–24 [56]. Quality of life will be measured by the disease-specific Hamburg Quality of Life in MS Scale (HAQUAMS) [57] and the EQ-5D [58]. While the EQ-5D showed excellent reliability, it showed a lack of content validity in MS by missing certain domains that were important to the disease and difficulties in differentiating between different levels of disability [59]. The HAQUAMS demonstrated validity and reliability [57, 60]. As a control parameter, we will measure anxiety and depression using the Hospital Anxiety and Depression Scale (HADS) [61] (Table 1).

**Table 1:** Study assessment

Instrument	Measurement time points											
	Screening baseline		Allocation	Post allocation								
Month	t_**−1**_	t_**0**_		t_**1**_	t_**2**_	t_**3**_	t_**4**_	t_**5**_	t_**6**_	t_**7**_	t_**8**_	t_**x**_
	− 1	0		3	6	9	12	15	18	21	24	X
Eligibility screen	X											
Informed consent	X											
Sociodemographic data	X	X										
Randomisation			X									
Relapse history				X	X	X	X	X	X	X	X	X
Extra questions in case of a relapse												
Relapse questions				(X)	(X)	(X)	(X)	(X)	(X)	(X)	(X)	(X)
Decision autonomy and satisfaction				(X)	(X)	(X)	(X)	(X)	(X)	(X)	(X)	(X)
CPS relapse				X								
PAM and empowerment scale		X		X			X				X	X
HAQUAMS		X					X					
EQ-5D		X		X			X				X	X
HADS		X					X					
PBMS relapses				X			X					
UNDS		X					X	X	X	X	X	X
Relapse risk knowledge				X			X					
Physician visit including EDSS		X					X					
Health economic parameters		X		X	X	X	X	X	X	X	X	X

t_-1_ = before enrolment; t_0_ = before randomization; t_1_ = month 3; t_2_ = month 6; t_3_ = month 9; t_4_ = month 12; t_5_ = month 15; t_6_ = month 18; t_7_ = month 21, t_8_ = month 24; t_x_ = after the final participant reaches t_4_ (all participants, who have not reached t_8_); (x): in case of relapse

*CPS* Control Preferences Scale, *EDSS* Expanded Disability Status Scale, *HADS* Hospital Anxiety and Depression Scale, *HAQUAMS* Hamburg Quality of Life in MS Scale, *PAM* Patient Activation Measure, *PBMS* Planned Behaviour in MS Scale, *UNDS* United Kingdom Neurological Disability Scale

#### Health economic outcomes

The objective of the health economic evaluation is to determine the efficiency of the intervention by comparing the cost and outcome of the intervention group to the cost and outcome of the control group. A cost-effectiveness (CEA) and a cost-utility analysis (CUA) will be carried out from the perspective of the German statutory health insurance and the society. The effect measure used in the CEA will be the primary outcome of the main trial, i.e. the proportion of untreated or orally treated relapses during the follow-up of at least 12 and at most 24 months. With respect to the CUA, quality-adjusted life years (QALYs) are calculated based on the EQ-5D-5L and evaluated with the German tariff to receive population-based utilities [58] during the follow-up of at least 12 and at most 24 months. Direct costs associated with the intervention as well as costs resulting from the consumption of health-related goods and services [62] (contacts to health care providers, hospital stays, therapists contacts, medical aids, and MS-related medication) and indirect costs due to productivity losses (sick leave days, disability pension) are considered. A questionnaire based on a standardised instrument [63] will be used to record the health care consumption of study participants.

### Participant timeline {13}

**Fig. 3:**
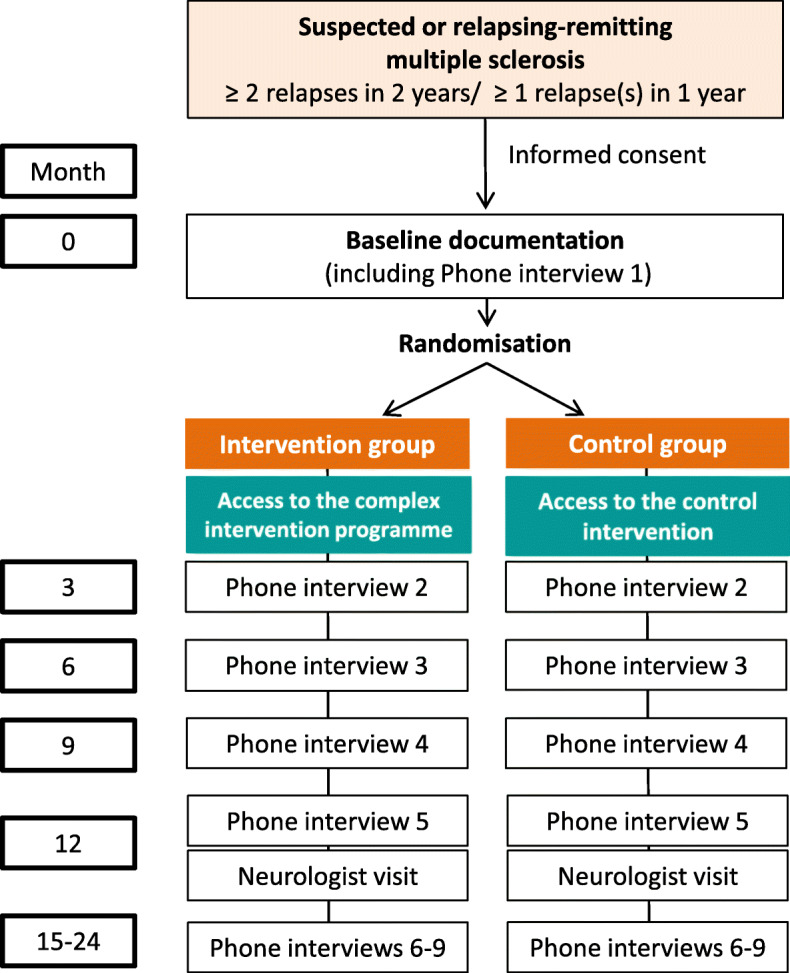
Study flow

For a description of the trial flow, see Fig. 3. After giving their informed consent, participants’ contact data (address, e-mail, telephone) will be forwarded to the study centre (UKE) via a secure communication platform or phone. After this, baseline data will be collected and group assignment will be performed by a block randomisation procedure within the monitoring platform secuTrial®. After successful randomisation within 30 days after baseline assessment, PwMS will receive access (login) details to the intervention or control platform. Since the intervention tools will be offered online, patients will be free to choose when and where they want to login.

Participants will be monitored for at least 12 and up to 24 months (on average 18 months). Every 3 months, measures are recorded by a standardised phone interview executed by the coordinating study centre (see Fig. 3).

Recruitment will be completed after approximately 15 months. The trial will end as soon as 81 relapses have been documented and the last participant has reached 12 months of follow-up. All participants, who have not reached 24 months of follow-up, will be called for a final phone interview.

### Sample size {14}

The unit of analysis for the primary endpoint is the occurrence of relapses. Eighty-one relapses per group yield a power of 85% at a two-tailed significance level of 5% given proportions of 78% and 56% of orally treated or non-treated relapses in IG and CG, respectively, as observed in the previous study [17]. It is expected that this relapse rate can be observed in a total of 170 patients with 1 to 2 years follow-up, corresponding to an annual relapse rate of 0.64. The dropout rate is expected to be about 10%, as in the previous study. Therefore, 188 participants will be randomised.

#### Sample size recalculation

Some of the assumptions including the overall relapse rate and the independence of the relapses will be checked in a sample size review based on non-comparative data. If indicated, e.g. because the relapse rate is lower than expected, the number of participants might be increased to a maximum of 280. As people may experience more than 2 relapses, we will analyse if management decisions differ between these events. If not, only the first relapse in any patient will be analysed. A primary recruitment period of 9 months is assumed with approx. 9–18 patients per practice or clinic (approx. 5 clinics, 12 practices) with 1–2 patients per month. With an increase to 280 participants, recruitment will be extended to 12 months.

### Assignment of interventions: allocation

#### Sequence generation {16a}

Eligible study participants will be randomised into the IG or to the CG (1:1 allocation ratio) stratified by the centre through a computer-generated system in secuTrial®. This will be done in blocks. The block sizes will not be disclosed.

#### Concealment mechanism {16b}

The randomisation will be automatically performed in secuTrial®. The menu button “Randomisation” is only visible for the person who has the right of randomisation (central study nurse). A participant can only be randomised once. Randomisation will follow within 30 days after inclusion.

#### Implementation {16c}

Participants will be enrolled in the participating centres. Randomisation follows automatically after the central study nurse in Hamburg pushed the menu button in secuTrial®.

### Assignment of interventions: blinding

#### Who will be blinded {17a}

The study will be conducted as an investigator and participant blinded trial. Participating physicians as well as MS centres, in general, will not be provided with any information about group assignment of participants. Blinding of the trial participants is pursued, but only possible to a limited extent. Furthermore, it cannot be prevented that patients discuss the intervention contents with their physician. Thus, participants and neurologists might realise their group assignment. While blinding in complex educational interventions including a webinar is virtually not possible, the only strategy to increase the similarity of groups is to have an active control group, which we aim for with the optimised standard care group. Furthermore, the outcome assessors are blinded.

#### Procedure for unblinding if needed {17b}

We do not assume that unblinding will be necessary due to the nature of the intervention.

## Data collection and management

### Plans for assessment and collection of outcomes {18a}

Data will be obtained at different time points using paper-based questionnaires. First of all, informed consent and patient-related contact data will be obtained from interested PwMS in the MS centres and a copy will be sent to the study centre (UKE). This transfer of contact details aims to minimise the effort of care-providers and to have maximum overview and control of trial conduct and data acquisition at the central study centre. Baseline data will be obtained by the participating MS centres before randomisation. They will note the pseudonym created by secuTrial® on the questionnaires. From this point in time, the study centre (UKE) will take over the handling of the participants (phone interviews, mailing of pseudonymised questionnaires and other contacts). To secure follow-up data, study participants will be contacted by the study centre via phone within the first 2 weeks to secure communication lines and to perform the phone interview. Blinded study assistants will be trained for this interaction. Trial data will be collected throughout the course of the study as well as during the last follow-up to examine the intervention effect on the study outcomes. However, beyond 3-monthly phone interviews, major assessments will be performed only at baseline, after 3 and after 12 months. Study participants receive prepaid envelopes to send the questionnaires back to the study centre in Hamburg.

### Plans to promote participant retention and complete follow-up {18b}

For adherence to the intervention see 11c. Participants in both groups will be contacted by phone every 3 months. For adherence to the completion of questionnaires, study participants will be asked to fill in the forms within 1 week. In case of missing data, study participants will be contacted and reminded about the completion by a member of the coordinating centre by e-mail or telephone. Participants, who did not participate in the study or control intervention, will be followed over the entire study period.

#### Patient withdrawal

Study participants may leave the study at any time and may withdraw consent to study participation without negative consequences. Reasons for discontinuation will be asked for and, if provided, recorded.

### Data management {19}

All data relevant to the study will be entered in secuTrial® and provided online. Medical staff of centres, as well as the central study team (defined health researchers and study assistants), can enter data. All data filled in paper-based will be transferred into secuTrial® and will be controlled by another member of the study team.

The preparation of the declaration of consent (including voluntariness) and the handling of all data collected within the scope of the study will be carried out in accordance with the recommendations of the Ethics Committee of the University of Lübeck and the EU General Data Protection Regulation (Datenschutz-Grundverordnung, DSGVO).

Data protection concerns regarding the intervention platform will be met by securing a protected web platform. The intervention programme, as well as the control programme, will be provided via a secure online platform that meets all legal requirements (e.g. encryption, certificates). The platform developed by GAIA has already been successfully used in several international studies [64] and is currently used in a multinational phase-3 study on depression management in MS.

All trial visit data will be captured and processed through the IT platform secuTrial® of the (MS Register of the DMSG) maintained by the MS Forschungs- und Projektentwicklungs-gGmbH (MSFP) in Hannover, who will be unaware of participants’ allocation and identifying data. Also, login data for participating centres (study nurses, physicians) will be provided via secuTrial®.

The secured chat room is provided via MS Connect, which is hosted by the DMSG (https://www.msconnect.de/Datenschutz). Access to an institutional application of Cisco WebEx, located at the UKE, will be provided by a member of the study team in Hamburg.

All electronic and paper-based data will be stored at the Institute of Neuroimmunology and Multiple Sclerosis at the University Medical Centre Hamburg-Eppendorf for a maximum period of 10 years and will be destroyed subsequently. In case of revoked consent, pseudonymised data will be anonymised and used in this form. A deletion of already anonymised data is not possible.

### Confidentiality {27}

All patient-related information will be pseudonymised to secure patient protection. However, all participating MS centres will have a securely stored list with names and assigned pseudonyms.

All study data will be used and evaluated pseudonymously by the members of the coordinating centre and consortium partners involved. The publication of the study results and the provision of the data in an online resource will only take place in an anonymised, e.g. aggregated form. Study participants will be informed about the results of the survey through a publication of the results on the DMSG website after the completion of the study.

Data transfer from MSFP, where the database is handled to the study centre in Hamburg, will be handled by the mailing of encrypted USB sticks with pseudonymised data in Excel or IBM SPSS format. Data will be secured on a protected computer at the INIMS, UKE. For trial analyses, validity-checked data will be transferred in the same way to the study statistician in Göttingen. As a backup, the participating MS centres will send paper-based data material to the study centre by regular mail.

### Plans for collection, laboratory evaluation and storage of biological specimens for genetic or molecular analysis in this trial/future use {33}

Not applicable as no biological specimens were collected as part of this trial.

## Statistical methods

### Statistical methods for primary and secondary outcomes {20a}

The primary endpoint is evaluated using a generalised linear model with mixed effects and logit link function. Subject-specific effects are modelled as random, whereas the intervention group (IG vs. CG) and study centre are included as fixed effects in the model. The intervention effect is reported as odds ratio (OR) with 95% confidence interval (CI) and *p* value testing the null hypothesis of no intervention effect (i.e. OR = 1). Longitudinal assessments of the quality of life and impairment are analysed employing Gaussian linear models for repeated measures (so-called mixed model for repeated measurements (MMRM)) with the intervention group (IG vs. CG), time, intervention-by-time interaction and study centre as factors and baseline score as a covariate. The error terms are assumed to follow a multivariate normal distribution with unstructured covariance. Least squares mean changes from baseline will be reported for both groups with 95% CI as well as the difference between the least-squares intervention group means (IG vs. CG) with 95% CI and *p* value testing the null hypothesis of no treatment effect.

#### Health economic analysis

To determine the efficiency of the intervention, a cost-effectiveness analysis (CEA) is performed in terms of additional costs per additional patient gained with untreated or orally treated relapse. In addition, a cost-utility analysis (CUA) will be carried out, which aims to calculate the additional costs required for an additional quality-adjusted life year (QALY). While the former yields the incremental cost-effectiveness ratio (ICER), the latter estimates the incremental cost-utility ratio (ICUR). ICER and ICUR are calculated similarly as the ratio of the difference in mean costs and difference in the mean outcomes between the intervention and control groups. Productivity losses will be estimated using the human capital approach [65]. Due to the short study period, no discounting of the effects and costs is planned. 95% confidence intervals for the outcome of the analyses will be calculated non-parametrically using bootstrap procedures [65]. Univariate and probabilistic sensitivity analyses will be performed, and cost-effectiveness acceptance curves will be executed to take into account uncertainty [66].

### Interim analyses {21b}

There will be no planned interim analysis that would require any adjustment of the significance level (critical value). However, a sample size review based on non-comparative data will be carried out (see the “Sample size {14}” section).

### Methods for additional analyses (e.g. subgroup analyses) {20b}

In subgroup and regression analyses, effects of age, gender, level of education, centre and level of impairment will be explored.

The process evaluation including the analyses will be described in an additional publication.

### Methods in analysis to handle protocol non-adherence and any statistical methods to handle missing data {20c}

All PwMS will be analysed in the group they were randomised to following the intention-to-treat principle. Early study discontinuations will be treated as an independent right censoring in the primary analysis. In case of substantial or differential study discontinuations, the validity of the independent censoring assumption will be explored in shared random effects models of the primary endpoint and time to study discontinuation. To handle missing data in baseline variables or follow-up assessments, multiple imputation models will be applied. For the knowledge questionnaires, we will follow questionnaire specific guidance to impute missing data. All details of the statistical analyses including definitions of analysis populations will be prespecified in the statistical analysis plan.

### Plans to give access to the full protocol, participant level-data and statistical code {31c}

Information will be provided on request.

## Oversight and monitoring

### Composition of the coordinating centre and trial steering committee {5d}

The steering committee is composed of experts and important stakeholders in the field of multiple sclerosis. The committee will meet via monthly telephone conferences and at the yearly meetings of the Deutsche Gesellschaft für Neurologie (DGN) to review the progress of the study and to make decisions within the framework of the study if necessary. All steering committee members must agree to the final protocol before publication. The steering committee is represented by the following members:

- Prof. Dr. Christoph Heesen, Medical Centre Hamburg-Eppendorf (steering committee chair)
- Ass.-Prof. Dr. Anne Rahn, Medical Centre Hamburg-Eppendorf
- Prof. Dr. Tim Friede, University Medical Centre Göttingen
- Alexander Stahmann, MS Forschungs- und Projektentwicklungs-gGmbH
- Dr. Jutta Scheiderbauer, MS Stiftung Trier

### Composition of the data monitoring committee, its role and reporting structure {21a}

There will be a formal external independent monitoring by CTC North GmbH & Co. KG (https://www.ctc-north.com/en.html?no_cache=1). The monitoring includes site visits of the central study centre in Hamburg to review informed consents and perform remote checks of the online database secuTrial® for data consistency and quality. A detailed monitoring plan is available in the German language on request.

### Adverse event reporting and harms {22}

As relevant adverse events are unlikely, no stopping rules will be applied. Nevertheless, safety measures are applied to control for anxiety, depression and disease-specific quality of life. Furthermore, standard disease monitoring parameters will be collected (e.g. relapse rate, disability status) and discussed by the steering committee.

We consider the specific risks for participating PwMS to be very low. No negative effects on the quality of life of PwMS as well as disability or other undesired events due to omitted or oral steroid administration are to be expected as previous studies [32] showed. It is more likely that there will be positive effects for trained PwMS in terms of more autonomous decision-making and differentiated use of steroids [32]. Participants (IG) will be informed about potentially dangerous side effects of steroid therapies and their early detection by an information sheet. To assist the physician in assessing whether oral medication is acceptable, participants (IG) will be issued a certificate with documented decision-making knowledge. As part of this study, all study participants will be contacted by phone every 3 months. This will also allow individual risk identification and the initiation of appropriate measures if required.

Nonetheless, it could be possible that some participants feel harassed or pressured by the intervention or the permanent contact attempts. To detect possible adverse events, PwMS and physicians will be asked by questionnaires throughout the study as part of the process evaluation. Since the programme is accessed from home, there is little organisational and time expenditure.

### Frequency and plans for auditing trial conduct {23}

Not applicable as there are no planned audits.

### Plans for communicating important protocol amendments to relevant parties (e.g. trial participants, ethical committees) {25}

Approval for protocol modifications and amendments will be sought for from the ethical committees at the University of Lübeck and reported to all relevant ethical committees. All changes will be noted in the study registration.

## Dissemination plans {31a}

This study protocol and study results will be published in major journals to disseminate the study results. In addition, all trial results will be communicated at scientific conferences and meetings (e.g. at the yearly DGN congress) by the investigators and presented on the DMSG website and other relevant patient websites.

Authorship will be shared between persons involved in the study following the current guidelines of the International Committee of Medical Journal Editors (ICMJE). Professional writers and persons not directly involved in the study will not be granted authorship.

## Discussion

The proposed RCT aims to assess the effectiveness of a web-based decision support programme concerning relapse management in MS in Germany. As this intervention is associated with potential structural management changes as for example the possibility of oral steroid management, the trial is accompanied by a thoroughly developed mixed-methods process evaluation to identify facilitating factors and barriers to the implementation of the intervention programme. The process evaluation will be described in an additional protocol. This study is innovative in several respects. In addition to the evaluation of the feasibility and the effectiveness of a web-based programme, it can provide initial insights into the cost-benefit ratio of online interventions in PwMS. Beyond that, the fit between the technology (patient decision aid), users and context factors will be explored by a process evaluation. A web-based programme will meet the request of PwMS for verified online information [28] and would be both resource-efficient and easily accessible. Reflecting the actual COVID-19 pandemic, the developed complex intervention could serve as a prototypical example for providing PwMS with comprehensive up-to-date information and support for treatment decision-making without a clinical visit and therefore reducing the risk of an infection during a disabling relapse [66]. It is expected that the programme will positively change patients’ relapse management and strengthen their autonomy and participation.

## Trial status

This is the protocol version 2.3 from 14 January 2021. Recruitment started in February 2020 and will end in December 2021 if recruitment is not prolonged after the planned interim analysis.

## Abbreviations

CG: Control group
CPS: Control Preference Sale
DGN: Deutsche Gesellschaft für Neurologie (German Society of Neurology)
DMSG: Deutsche Multiple Sklerose Gesellschaft (German MS Self-help Society)
EBPI: Evidence-based patient information
EDSS: Expanded Disability Status Scale
HADS: Hospital Anxiety and Depression Scale
HAQUAMS: Hamburg Quality of Life in MS Scale
ICER: Incremental cost-effectiveness ratio
ICUR: Incremental cost-utility ratio
HARAS: Hamburg Relapse Assessment Scale
IV: Intravenous
MMRM: Mixed model for repeated measurements
MRC: Medical Research Council
MS: Multiple sclerosis
MSFP: MS Forschungs- und Projektentwicklungs-gGmbH
PAM: Patient Activation Measure
PBMS: Planned Behaviour in MS Scale
PwMS: People with multiple sclerosis
QALY: Quality-adjusted life year
RRMS: Relapsing-remitting multiple sclerosis
RCT: Randomised controlled trial
UK: United Kingdom
UNDS: United Kingdom Neurological Disability Scale
