# Correction to: Development and evaluation of an interactive web-based decision-making programme on relapse management for people with multiple sclerosis (POWER@MS2)—study protocol for a randomised controlled trial

**DOI:** 10.1186/s13063-021-05152-5

**Published:** 2021-03-08

**Authors:** Anne Christin Rahn, Lisa Wenzel, Andrea Icks, Alexander Stahmann, Jutta Scheiderbauer, Kristina Grentzenberg, Markus Vomhof, Joseph Montalbo, Tim Friede, Christoph Heesen, Sascha Köpke

**Affiliations:** 1 Institute of Neuroimmunology and Multiple Sclerosis, University Medical Center Hamburg-Eppendorf, Hamburg, Germany; 2 Department of Health Services Research, Carl von Ossietzky University Oldenburg, Oldenburg, Germany; 3 Institute of Nursing Science, University Hospital Cologne and Faculty of Medicine, University of Cologne, Cologne, Germany; 4 Institute for Health Services Research and Health Economics, Centre for Health and Society, Heinrich Heine University Düsseldorf, Düsseldorf, Germany; 5 MS Forschungs- und Projektentwicklungs-gGmbH, Hannover, Germany; 6 Stiftung für Selbstbestimmung und Selbstvertretung von MS-Betroffenen, Trier, Germany; 7 Department of Medical Statistics, University Medical, Göttingen, Germany; 8 Department of Neurology, University Medical Center Hamburg-Eppendorf, Hamburg, Germany

**Correction to: Trials (2021) 21:139**

**https://doi.org/10.1186/s13063-021-05059-1**

Following publication of the original article [1], we were notified that the legend for Table 1 was missing.

The original article has been corrected.
